# The Arabidopsis effector-triggered immunity landscape is conserved in oilseed crops

**DOI:** 10.1038/s41598-022-10410-w

**Published:** 2022-04-20

**Authors:** Clare Breit-McNally, Darrell Desveaux, David S. Guttman

**Affiliations:** 1 Department of Cell and Systems Biology, University of Toronto, Toronto, ON Canada; 2 Centre for the Analysis of Genome Evolution and Function, University of Toronto, Toronto, ON Canada

**Keywords:** Natural variation in plants, Plant domestication, Plant immunity

## Abstract

The bacterial phytopathogen *Pseudomonas syringae* causes disease on a wide array of plants, including the model plant *Arabidopsis thaliana* and its agronomically important relatives in the Brassicaceae family. To cause disease, *P. syringae* delivers effector proteins into plant cells through a type III secretion system. In response, plant nucleotide-binding leucine-rich repeat proteins recognize specific effectors and mount effector-triggered immunity (ETI). While ETI is pervasive across *A. thaliana*, with at least 19 families of *P. syringae* effectors recognized in this model species, the ETI landscapes of crop species have yet to be systematically studied. Here, we investigated the conservation of the *A. thaliana* ETI landscape in two closely related oilseed crops, *Brassica napus* (canola) and *Camelina sativa* (false flax). We show that the level of immune conservation is inversely related to the degree of evolutionary divergence from *A. thaliana*, with the more closely related *C. sativa* losing ETI responses to only one of the 19 *P. syringae* effectors tested, while the more distantly related *B. napus* loses ETI responses to four effectors. In contrast to the qualitative conservation of immune response, the quantitative rank order is not as well-maintained across the three species and diverges increasingly with evolutionary distance from *A. thaliana*. Overall, our results indicate that the *A. thaliana* ETI profile is qualitatively conserved in oilseed crops, but quantitatively distinct.

## Introduction

Gram-negative phytopathogenic bacteria, such as *Pseudomonas syringae* deliver type III secreted effector proteins (effectors hereafter) into host cells through a type III secretion system, where they function to suppress basal plant immunity and promote pathogen growth^1,2^. However, plant nucleotide-binding leucine-rich repeat (NLR) proteins can recognize the presence or activity of certain effectors and mount a robust immune response called effector-triggered immunity (ETI), which limits pathogen proliferation^3,4^. A systematic analysis of the ETI landscape of the model plant *Arabidopsis thaliana* against *P. syringae* revealed that 19 of 70 families of *P. syringae* effectors trigger ETI in the Col-0 ecotype of *A. thaliana*^5^. It was further revealed that ETI is remarkably pervasive in this plant-pathogen interaction, with nearly all analyzed *P. syringae* strains carrying at least one effector that has the potential to elicit ETI in *A. thaliana*. This suggests that ETI plays an important role in contributing to broad-spectrum disease resistance; however, it remains to be determined whether such a prominent ETI landscape exists beyond Arabidopsis.

*A. thaliana* is a small, annual weedy plant that belongs to the Brassicaceae, or mustard family, which also includes many important food crops such as radish, kale, and broccoli as well as oilseed crops such as *Brassica napus* (canola) and *Camelina sativa* (false flax)^6^. *B. napus* has an extensive breeding history as one of the world’s most important oilseeds, while *C. sativa* is an important emerging crop. *C. sativa* is in the tribe (i.e., subfamily) Camelineae, which includes *A. thaliana*, and these two species are believed to have diverged approximately 8 million years ago^7^. *B. napus*, on the other hand, is in the tribe Brassiceae, which is believed to have diverged from the ancestor of *A. thaliana* approximately 23 million years ago^7^. Consequently, these three species provide an interesting continuum, from the relatively divergent and highly cultivated *B. napus*, to the relatively closely related and recently cultivated *C. sativa,* to the ‘wild’ model species *A. thaliana*^6,8–10^. Despite both being crop species, *B. napus* and *C. sativa* offer similar advantages for researchers as the model plant *A. thaliana*, such as the availability of reference genomes^11,12^ and their amenability to genetic manipulation^13–19^.

Genome-wide comparative analyses of NLRs have revealed a high degree of NLR diversity within Brassicaceae^20–27^. For instance, compared to approximately 165 NLRs in the diploid *A. thaliana*, there are approximately 464 NLR-encoding genes in the tetraploid *B. napus*, which may reflect an expanded ETI landscape relative to *A. thaliana*^24^. The NLR repertoire of the hexaploid *C. sativa* has yet to be characterized. Nevertheless, orthologs of characterized NLRs from *A. thaliana* can be identified in both *C. sativa* and/or *B. napus*^25^. For instance, two copies of the NLR RPM1, which is required for the recognition of *P. syringae* effectors AvrB1 and AvrRpm1, are known to occur in *B. napus*^28^. Both *B. napus* and *C. sativa* also possess orthologs of ZAR1^29^, which is required for the recognition of at least five distinct families of *P. syringae* effectors in *A. thaliana*: HopZ1, HopF1, HopBA1, HopO1, and HopX1^5,30,31^. While the presence of NLR orthologs may indicate the conservation of ETI responses in close relatives of *A. thaliana*, this has yet to be systematically validated in Brassicaceous crops such as *B. napus* and *C. sativa*.

Here, we surveyed the conservation of ETI responses between *A. thaliana* and its two agronomically important Brassicaceous relatives, *B. napus* and *C. sativa*, by first establishing pathology assays on *B. napus* and *C. sativa*, and then screening these plant species with the 19 *P. syringae* effector families that elicit ETI in *A. thaliana*. We show that the *P. syringae* pathovar tomato DC3000 (*Pto*DC3000) can be adapted to study ETI on *B. napus* and *C. sativa*, and that 15 and 18 of the 19 ETI responses are conserved in *B. napus* and *C. sativa*, respectively. Our results indicate that the *A. thaliana* ETI responses are retained in oilseed crops, suggesting that domestication has not substantially compromised their ETI potential.

## Results

### Establishing *P. syringae *pathology assays on *B. napus *and *C. sativa*

**Figure 1:**
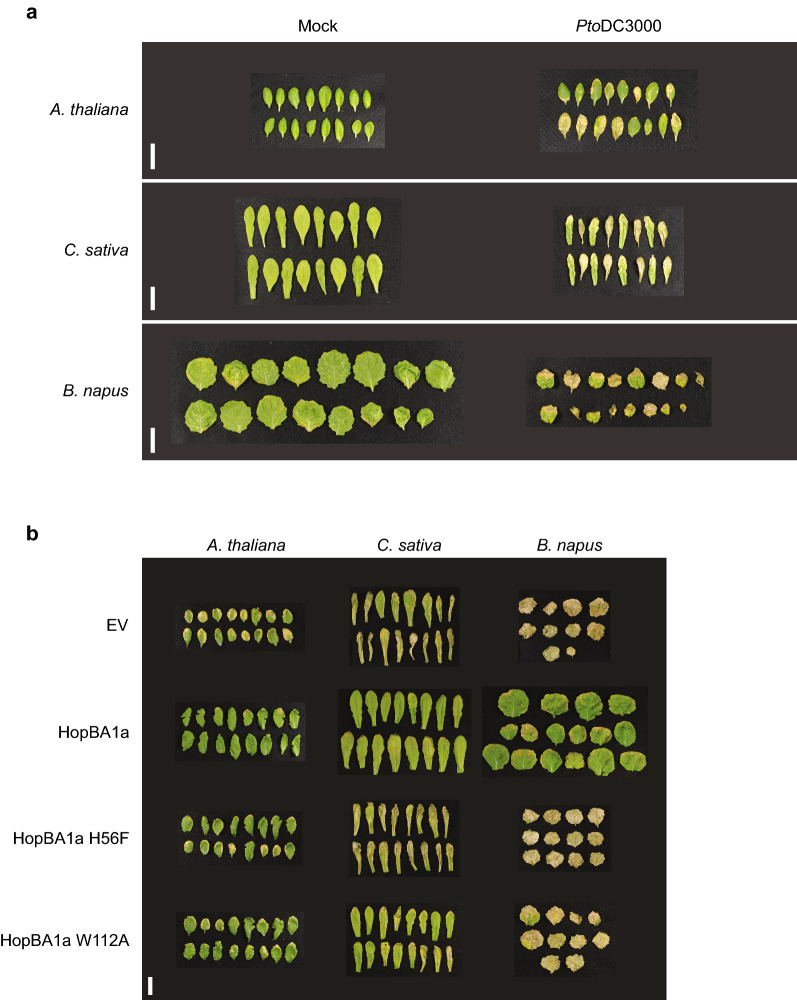
Establishing *P. syringae* pathology assays on *B. napus* and *C. sativa*. (**a**) *P. syringae* pv. tomato strain DC3000 (*Pto*DC3000) causes disease symptoms on *B. napus* and *C. sativa* similar to those on *A. thaliana. A. thaliana* Col-0, *B. napus* var. Topas, and *C. sativa* var. DH55 were spray inoculated with either a mock treatment of 10 mM MgSO_4_ 0.04% Silwet (Mock) or *Pto*DC3000 at an OD_600_ = 1. *A. thaliana* was sprayed at 4 weeks old. *B. napus* and *C. sativa* were sprayed at 2 weeks old. Images show 2 leaves per plant. Photographs were taken at 6 days post-infection. Scale bar = 3 cm. (**b**) HopBA1a elicits ETI in *B. napus* and *C. sativa*. Representative photographs of chlorotic symptoms on *A. thaliana* Col-0, *B. napus* var. Topas, and *C. sativa* var. DH55 infected with *Pto*DC3000 expressing an empty vector (EV), HopBA1a, HopBA1a H56F, or HopBA1a W112A. Plants were spray inoculated as described above. Images show 2 leaves per plant. Photographs were taken at 6 days post-infection. Scale bar = 3 cm.

We first sought to identify a strain of *P. syringae* capable of causing disease on *B. napus* and *C. sativa*. In *A. thaliana* Col-0, spray inoculation of the highly virulent strain *Pto*DC3000 leads to chlorotic disease symptoms^32–34^. Similarly, spray inoculation of *Pto*DC3000 on *B. napus* and *C. sativa* resulted in distinctive and consistent chlorotic symptoms on both plant species to a similar extent as on *A. thaliana* Col-0 (Fig. 1a). Interestingly, disease symptoms on *B. napus* and *C. sativa* were associated with a more pronounced stunting of plant growth relative to *A. thaliana* (Fig. 1a).

To determine whether *B. napus* and *C. sativa* could recognize *P. syringae* effectors and mount an ETI response, we spray inoculated *B. napus* and *C. sativa* with *Pto*DC3000 strains expressing an effector from each of the families that trigger ETI in *A. thaliana* Col-0^5^ and assessed their ETI eliciting potential relative to a virulent negative control (*Pto*DC3000 carrying a pBBR1-MCS2 empty vector). In both *B. napus* and *C. sativa*, HopBA1a triggered a significant reduction in both disease symptoms and *in planta* bacterial growth compared to the empty vector control, indicating that it triggers a strong ETI response in these two plant species (Fig. 1b, Supplemental Figure 1). We further tested two point mutations in HopBA1a (H56F and W112A) that are known to abolish ETI-associated hypersensitive responses in the *A. thaliana* ecotype Ag-0^35^ and found that they likewise restored disease symptoms and bacterial growth in *B. napus* and *C. sativa*, suggesting a similar recognition mechanism based on the interaction interfaces or catalytic activity that these residues confer to HopBA1a (Fig. 1b, Supplemental Figure 1). Overall, these results demonstrate that *Pto*DC3000 can grow and cause disease symptoms on *B. napus* and *C. sativa* and that ETI can suppress the virulence associated outcomes of *P. syringae* infection. This pathosystem could therefore be used to compare the ETI landscapes of these oilseed crops with that of *A. thaliana*.

### Qualitative conservation of the *A. thaliana *ETI landscape across *B. napus *and *C. sativa*

**Figure 2:**
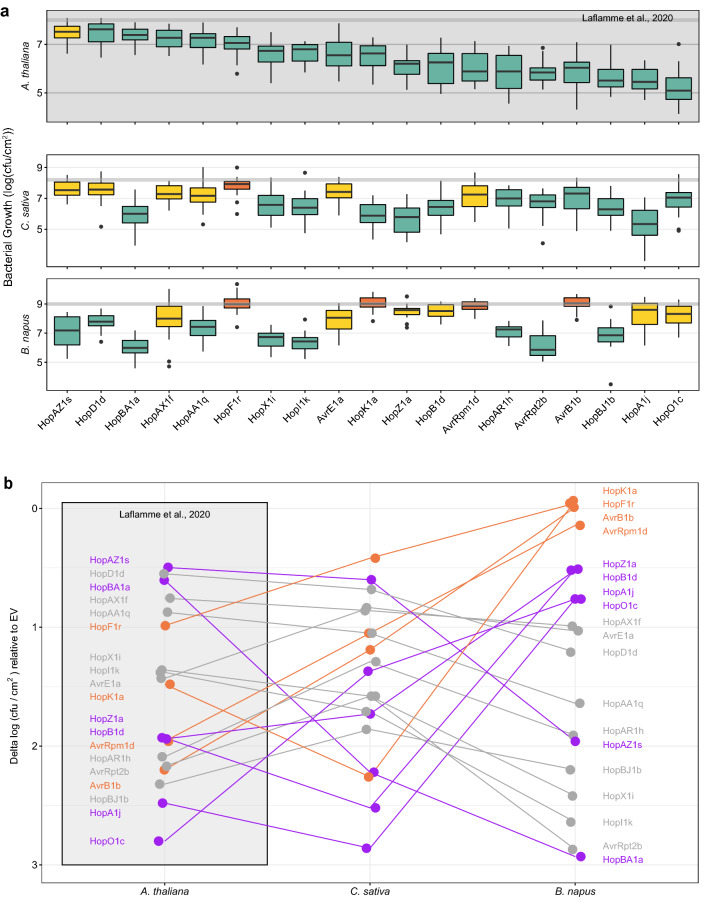
Conservation of the *A. thaliana* Col-0 ETI responses in *C. sativa* var. DH55 and *B. napus* var. Topas. (**a**) Growth assays of *Pto*DC3000 expressing 19 ETI eliciting effectors identified in^5^ normalized to the empty vector (EV) across assays for each species. *A. thaliana* Col-0 growth assay data is from Laflamme et al., 2020. The horizontal grey line across each plot represents the normalized mean of EV controls between assays. Green boxes represent the effectors that consistently caused a significant reduction in bacterial growth compared to the EV (ANOVA with post-hoc Tukey–Kramer HSD test, *P* < 0.05). Yellow boxes represent the effectors that led to inconsistent reductions in bacterial growth (not significantly different from EV in at least one experimental replicate). Orange boxes represent the effectors that were not significantly different from EV in any replicate. Box and whisker plots show pooled data from three experiments (n = 7 or 8 plants / experiment). Boxes show the first quartile, median, and third quartile. Whiskers extend to the smallest, and largest values no further than 1.5 × interquartile range from the first and third quartiles, respectively. Outlying points are plotted individually as solid circles. Raw growth assay data is presented in Supplemental Figure 2. (**b**) ETI intensity rank order profiles for *A. thaliana* Col-0, *B. napus* var. Topas, and *C. sativa* var. DH55. The delta log cfu/cm^2^ values of the normalized means of each effector relative to EV are plotted ranging from 0.0 logs (no reduction in bacterial growth relative to EV) to 3.0 logs (largest reduction in bacterial growth corresponding with the strongest ETI responses). Lines connect the means of each effector across the three plant species. Labels represent effector names. Orange represents effectors that do not trigger ETI in *B. napus* or *C. sativa*. Purple represents effectors of interest with very different responses between plants. Grey represents effectors that show similar responses between plants.

To assess the conservation of ETI responses between *B. napus*, *C. sativa,* and *A. thaliana*, we screened through the 19 ETI eliciting effector families previously identified in *A. thaliana* Col-0^5^ (Table 1) by quantifying their ETI eliciting potential using bacterial growth assays (Fig. 2, Supplemental Figure 2). We normalized bacterial growth reductions induced by each effector to a virulent control (*Pto*DC3000::empty vector) and applied the same analysis to the *A. thaliana* Col-0 growth assay data from Laflamme et al. (2020) for comparison (Fig. 2a; Supplemental Figure 2)^5^. The *C. sativa* and *B. napus* spray inoculation resulted in a spectrum of immune phenotypes, which were broadly classified as: (1) strong ETIs, with a significant reduction in bacterial growth in all three replicates; (2) inconsistent ETIs, with a significant reduction in one or two of the three replicates; or (3) non-ETI, causing no significant reduction in bacterial growth in any replicate. In *C. sativa*, 12/19 effectors (63%) triggered strong ETI responses; 6/19 (32%) led to inconsistent ETI responses, and 1/19 effectors (5%) did not trigger ETI (Fig. 2a). In *B. napus*, 9/19 effectors (47%) triggered strong ETI responses, whereas 6/19 (32%) displayed inconsistent ETI phenotypes, and 4/19 effectors (21%) did not trigger an ETI response (Fig. 2a). ETI responses to the effectors AvrRpm1d, AvrB1b, and HopK1a, were lost in *B. napus*, while HopF1r-triggered ETI was lost in both *B. napus* and *C. sativa* (Fig. 2a)*.* These results suggest that the majority of ETI responses are conserved between *A. thaliana* Col-0 and its two close relatives, though the robustness of these ETIs differ considerably across plant species.

**Table 1:** ETI eliciting effectors used in this study.

Effector family	Effector allele	Strain	Accession	Locus
AvrB	AvrB1b	PgyICMP807	NZ_RBNZ01000352.1	
AvrRpm1	AvrRpm1d	PfiICMP7848	NZ_LJQJ01000610.1	
AvrRpt2	AvrRpt2b	Pla1188_1	NZ_RBPG01000101.1	
HopK	HopK1a	PbrICMP13650	NZ_LJPV01000582.1	
HopAR	HopAR1h	PmeN6801	NZ_LGLB01000021.1	
HopA	HopA1j	PacICMP9850	NZ_RBSM01000105.1	
HopF	HopF1r	Pac302273	NZ_GL385316.1	
HopZ	HopZ1a	PssA2	NZ_LGKU01000014.1	
HopO	HopO1c	PsyUSA007	NZ_AVDY02000338.1	
HopX	HopX1i	PdpICMP13052	NZ_RBRA01000108.1	
HopBA	HopBA1a	PsfICMP4996	NZ_RBSD01000159.1	
HopB	HopB1d	PsyCC1466	NZ_AVEM02000219.1	
AvrE	AvrE1a	PsvICMP13519	NZ_RBNW01000319.1	
HopAA	HopAA1q	PsyCC1416	NZ_AVEP02000280.1	
HopD	HopD1d	PgyICMP2185	NZ_RBRH01000243.1	
HopI	HopI1k	PafICMP5011	NZ_RBOK01000041.1	
HopAX	HopAX1f	PcdICMP12341	NZ_RBOV01000268.1	
HopAZ	HopAZ1s	PhoICMP7847	NZ_CP042804.1	PSYTB_RS09780
HopBJ	HopBJ1b	PsyCC1466	NZ_AVEM02000066.1

### Quantitative conservation of the *A. thaliana *ETI landscape across *B. napus *and *C. sativa*

To assess the relative strengths of ETI in *C. sativa* and *B. napus*, we compared the rank order of normalized bacterial growth based on calculated delta log cfu/cm^2^ values relative to the EV and applied the same analysis to the *A. thaliana* Col-0 growth assay data from Laflamme et al. (2020) for comparison^5^ (Fig. 2b, Table 2). Notably, HopBA1a triggered strong decreases in bacterial growth in *C. sativa* (rank 4 of 19 ETI responses) and *B. napus* (rank 1) but was among the weakest ETI elicitors in *A. thaliana* (rank 17)*.* HopAZ1s, which was among the weakest elicitors in *A. thaliana* (rank 19) and *C. sativa* (rank 18) led to a strong ETI response in *B. napus* (rank 6)*.* In contrast, the ETI responses to HopA1j, HopB1d, and HopZ1a were among the strongest in *A. thaliana* (rank 2, rank 8, and rank 9 respectively) and *C. sativa* (rank 1, rank 6, and rank 2 respectively) but were weak in *B. napus* (rank 13, rank 14, and rank 15 respectively). HopO1c was the strongest ETI response in *A. thaliana* (rank 1) but was among the weak ETI elicitors in *C. sativa* (rank 10) and *B. napus* (rank 12). The overall ranked (Spearman) correlation between *A. thaliana* and *C. sativa* was 0.47, while the ranked correlation between *A. thaliana* and *B. napus* was − 0.24, indicating that the divergence of ETI profiles increases with evolutionary distance from *A. thaliana* (Supplemental Figure 3). Overall, these results emphasize that although the majority of ETI responses are conserved across the three Brassicaceous species, the quantitative nature of their ETI profiles are distinct.

**Table 2:** Rank order of ETI responses in *A. thaliana*, *B. napus*, and *C. sativa*.

Effector	Rank order of ETI responses based on delta log cfu/cm^2^ values relative to EV^a^			Delta log cfu/cm^2^ relative to EV			Significance^b^	
	*At*	*Cs*	*Bn*	*At*	*Cs*	*Bn*	*At* vs. *Cs*	*At* vs. *Bn*
AvrB1b	4	12	17	2.20	1.19	− 0.010	*	***
AvrE1a	11	16	10	1.43	0.835	1.03	*	***
AvrRpm1d	7	14	16	1.96	1.05	0.143	**	***
AvrRpt2b	5	8	2	2.17	1.58	2.87		
HopA1j	2	1	13	2.48	2.86	0.762		***
HopAA1q	15	13	8	0.873	1.05	1.64		
HopAR1h	6	11	7	2.09	1.29	1.91	*	***
HopAX1f.	16	15	11	0.755	0.861	0.991		
HopAZ1s	19	18	6	0.496	0.600	1.96		
HopB1d	8	6	14	1.94	1.73	0.520		***
HopBA1a	17	4	1	0.604	2.22	2.93	***	***
HopBJ1b	3	5	5	2.32	1.86	2.20		**
HopD1d	18	17	9	0.550	0.682	1.21		
HopF1r	14	19	18	0.987	0.419	− 0.044	**	***
HopI1k	12	7	3	1.38	1.71	2.64		
HopK1a	10	3	19	1.48	2.26	− 0.066		***
HopO1c	1	10	12	2.80	1.37	0.763	***	***
HopX1i	13	9	4	1.36	1.58	2.42		
HopZ1a	9	2	15	1.93	2.52	0.511		***

At = *A. thaliana*, Cs = *C. sativa*, and Bn = *B. napus*.

^a^Rank order is based on normalized bacterial growth assay data presented in Fig. 2, with rank 1 being the strongest ETI response (largest reduction in bacterial growth) and rank 19 being the weakest ETI response (smallest reduction in bacterial growth).

^b^Significance is based on T-tests comparing normalized growth between *A. thaliana* and *C. sativa* and between *A. thaliana* and *B. napus.* T-test p-values were Bonferroni corrected for 19 × 2 = 38 tests. Bonferroni corrected p-values are indicated by 0.05 > * > 0.01 > ** > 0.001 > ***.

## Discussion

We have established *P. syringae* pathology assays on *B. napus* and *C. sativa* and found that the *A. thaliana* ETI landscape is well conserved in these two Brassicaceous oilseed crop species. Out of 19 representative effectors that elicit ETI in *A. thaliana* Col-0^5^, 18 elicited ETI in *C. sativa* and 15 elicited ETI in *B. napus* (Fig. 2a). Since *A. thaliana* is more closely related to *C. sativa* than *B. napus*^7^, the greater overlap between *A. thaliana* and *C. sativa* ETI responses may be reflective of a more similar arsenal of NLRs. In addition, the ETI profiles of *B. napus* and *C. sativa* may differ due to their different domestication histories. Further studies that leverage host diversity by surveying the ETI profiles across multiple accessions of the three plant species will be required to establish the full extent of ETI conservation.

We observed a difference in the patterns of qualitative and quantitative ETI responses across species that diverged with evolutionary distance from *A. thaliana*. While most of the ETI responses tested in our study are qualitatively conserved across *A. thaliana*, *B. napus*, and *C. sativa*, the quantitative magnitudes of these ETI responses differ considerably between the three species, with many weak and inconsistent responses in *B. napus* and *C. sativa* (Fig. 2b). From a qualitative perspective, the loss of ETI responses were nested and correlated with the evolutionary distance from *A. thaliana* (i.e., both species lost HopF1r, while only *B. napus* lost HopK1a, AvrRpm1d, and AvrB1b)*.* The patterns are not nearly as clear from a quantitative perspective, with seven and ten inconsistent or absent ETI responses in *C. sativa* and *B. napus*, respectively. Of these, only four are shared (Jaccard similarity = 0.31), including two of the four lost ETI responses of *B. napus*. This pattern can also be seen in the extensive shuffling of ETI rank order between *B. napus* and *C. sativa* using bacterial growth assays (Fig. 2b). Nevertheless, the overall quantitative ETI profile of *C. sativa* was more similar to *A. thaliana* than *B. napus* (Supplemental Figure 3, Table 2). This suggests that there are more complex genomic differences that govern ETI responses than simple gain and loss of NLRs, leading to a continuum of disease and immune phenotypes across evolutionarily related plant species^5^.

Overall, our results suggest that effector recognition is broadly conserved across the Brassicaceae, indicating the possible functional conservation of several important NLRs. For example, the *A. thaliana* NLR ZAR1 is required for the recognition of at least five families of *P. syringae* effectors (HopZ1, HopF1, HopBA1, HopO1, and HopX1)^5,30,31^. *ZAR1* is broadly conserved across angiosperms, and both *B. napus* and *C. sativa* possess several *ZAR1* orthologs^29^. In both *B. napus* and *C. sativa*, four ZAR1-mediated ETI responses (HopX1i, HopZ1a, HopBA1a, and HopO1c) are conserved (Fig. 2a). However, HopF1r-mediated ETI is lost in both *B. napus* and *C. sativa*. ZAR1 associates with receptor-like cytoplasmic kinases (RLCKs) called ZED1-related kinases (ZRKs) and PBS1-like kinases (PBLs) to mediate ETI^36^. The loss of HopF1r-triggered ETI could indicate that its respective ZRK, ZRK3^31^, is absent or non-functional in *B. napus* and *C. sativa*. However, ZRK3 is also required for ZAR1-mediated recognition of HopO1c in *A. thaliana*^36^, which can still elicit ETI in *B. napus* and *C. sativa*. The PBL kinase PBL27 is also required for HopF1r ETI, but not HopO1c ETI in *A. thaliana*^37^. We therefore hypothesize that PBL27 or another component of the ZAR1 ETI machinery necessary for HopF1r-triggered ETI in *A. thaliana* is absent or non-functional in *B. napus* and *C. sativa*. Alternatively, these ETI responses (or a subset of them) may be ZAR1-independent and mediated by convergent evolution of distinct NLR genes as observed in other crop species^38–45^.

While HopF1r-triggered ETI was the only ETI response lost in both *B. napus* and *C. sativa*, three additional ETI responses (to the effectors HopK1a, AvrRpm1d, and AvrB1b) were lost in *B. napus* (Fig. 2a). Our results agree with a previous study that found that *B. napus* does not recognize AvrB1 or AvrRpm1, despite possessing two copies of *RPM1*, the NLR responsible for recognizing these two effectors in *A. thaliana*^28^. In *A. thaliana,* HopK1a (also known as AvrRps4^46,47^) is recognized by two distinct pairs of NLRs, RPS4/RRS1^48,49^ and RPS4b/RRS1b^50^. *B. napus* possesses homologs of RPS4 and RRS1b, but not RRS1 or RPS4b. Improper pairings such as RPS4/RRS1b or RPS4b/RRS1 do not recognize HopK1a in *A. thaliana*^50^, perhaps explaining the lack of HopK1a recognition in *B. napus*.

While it is known that *C. sativa* possesses several orthologs of *ZAR1*^29^, and that *B. napus* possesses copies of both *ZAR1* and *RPM1*^28,29^, the presence of other important NLRs that are known to recognize *P. syringae* effectors in *A. thaliana* have not yet been confirmed in these two species. A BLASTP^51^ analysis of *RPM1*, *RPS2*, *RPS4*, *RRS1, RPS4b, RRS1b*, *RPS5*, *RPS6*, *ZAR1*, *CAR1*, *BAR1,* and *RBA1* in *B. napus* and *C. sativa* revealed the presence of putative homologs for all but three of these NLRs in *B. napus* and/or *C. sativa* (Supplemental Table 1), which may explain the conservation of ETI profiles in *B. napus* and *C. sativa*. Interestingly, *RBA1*, which recognizes HopBA1 in the Ag-0 ecotype of *A. thaliana*^35^*,* is the only NLR absent in both *B. napus* and *C. sativa*. As such, HopBA1a, which triggers strong ETI responses in both *C. sativa* and *B. napus* (Fig. 2) may be recognized by ZAR1 as observed in *A. thaliana* Col-0^5^.

This study is the first example of a comprehensive investigation of ETI conservation between an important model plant and two closely related crop species. Most of the *A. thaliana* ETI responses are retained in *B. napus* and *C. sativa*, suggesting that domestication has not severely compromised ETI potential. Further studies testing the remaining alleles within ETI eliciting families will reveal whether *A. thaliana*, *B. napus*, and *C. sativa* possess differential allele specificity. Further, it will be interesting to comprehensively assess the ETI potential of the *P. syringae *Type III Effector Compendium in *B. napus* and *C. sativa* to determine whether the high extent of NLR diversity within Brassicaceous crops underlies an expansive ETI landscape beyond what is captured in *A. thaliana*. Nevertheless, our identification of ETI responses in *B. napus* and *C. sativa* provides insight into the conservation of crop immunodiversity and can be used to guide the development of crop protection strategies in these two important oilseed crops.

## Materials and methods

### Plant materials

*A. thaliana* Col-0*, B. napus* var. Topas and *C. sativa* var. DH55 plants were grown in Sunshine Mix 1 soil at constant 22 °C, a light intensity of 150 μmol/m^2^s, and a 12-h photoperiod. *B. napus* and *C. sativa* plants were sprayed at two weeks old, and *A. thaliana* plants were sprayed at three to four weeks old.

### Spray infiltrations

*P. syringae* strains used in this study were previously described^5^. Prior to spray inoculation, bacterial strains were grown overnight at 28 °C on KB agar amended with 50 μg/ml rifampicin and 50 μg/ml kanamycin. Strains were re-suspended in 10 mM MgSO_4_ with 0.04% silwet surfactant L-77 and diluted to 1 × 10^9^ CFU/ml (OD_600_ = 1). Individual plants were sprayed with approximately 3 ml of inoculum using Preval pressurized sprayers.

### Growth assays

Bacterial growth assays were performed three days post-infection. Four leaf discs (1 cm^2^) from each plant were harvested and ground in 1 ml of 10 mM MgSO_4_ using a bead-beater. Serial dilutions were performed and 5 μL of each sample was plated on KB agar amended with 50 μg/ml rifampicin and incubated at 28 °C for 24 h, after which individual colonies were counted. To compare bacterial growth across individual assays, growth assay data was normalized to the empty vector (EV) for each species (mean of 8.0, 8.2, and 9.0 log cfu/cm^2^ for *A. thaliana* Col-0, *C. sativa,* and *B. napus*, respectively) (Fig. 2a). To determine the quantitative rank order of ETI responses in each species, the delta log cfu/cm^2^ values were calculated for each effector relative to the EV using the normalized growth assay data (mean EV log cfu/cm^2^ – mean effector log cfu/cm^2^) (Fig. 2b, Table 2).

## Supplementary Information

Supplementary Information 1.

Supplementary Information 2.

## Data Availability

The datasets generated and/or analyzed during the current study are available from the corresponding authors on reasonable request.
