# Supplementary material for: The Arabidopsis effector-triggered immunity landscape is conserved in oilseed crops
Source: Sci Rep. 2022 Apr 20;12:6534. doi: 10.1038/s41598-022-10410-w (PMC9021255; doi:10.1038/s41598-022-10410-w)
Supplement: Supplementary file 1 — Supplementary Information 1. [file 41598_2022_10410_MOESM1_ESM.pdf]

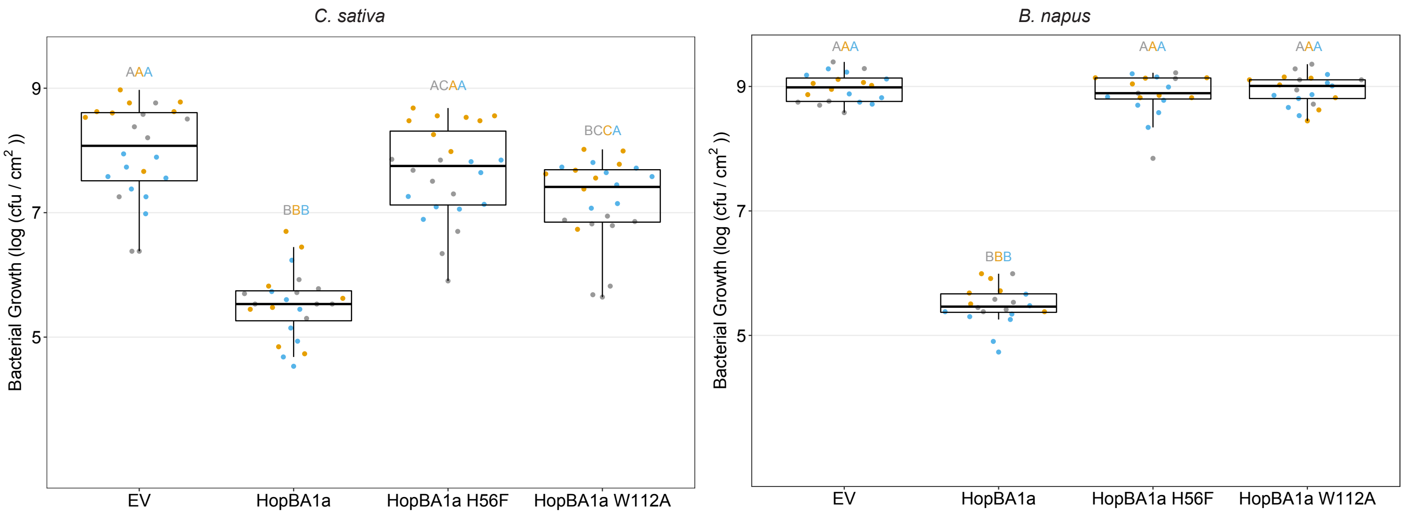

Supplemental Figure 1. HopBA1a elicits ETI in *B. napus* var. Topas and *C. sativa* var. DH55. Bacterial growth assays on *B. napus* and *C. sativa* infected with *Pto*DC3000 expressing an EV, HopBA1a, HopBA1a H56F, or HopBA1a W112A. Plants were spray inoculated with bacterial strains at an OD_{600} = 1 and growth assays were performed 3 days post-infection. Boxes show the first quartile, median, and third quartile. Whiskers extend to the smallest, and largest values no further than 1.5x interquartile range from the first and third quartiles, respectively. Dots indicate individual plants, and different colours represent single experiments (n = 5, 6, 7, or 8 plants / experiment). Letters indicate significance categories (ANOVA with post-hoc Tukey-Kramer HSD test, $P < 0.01$).

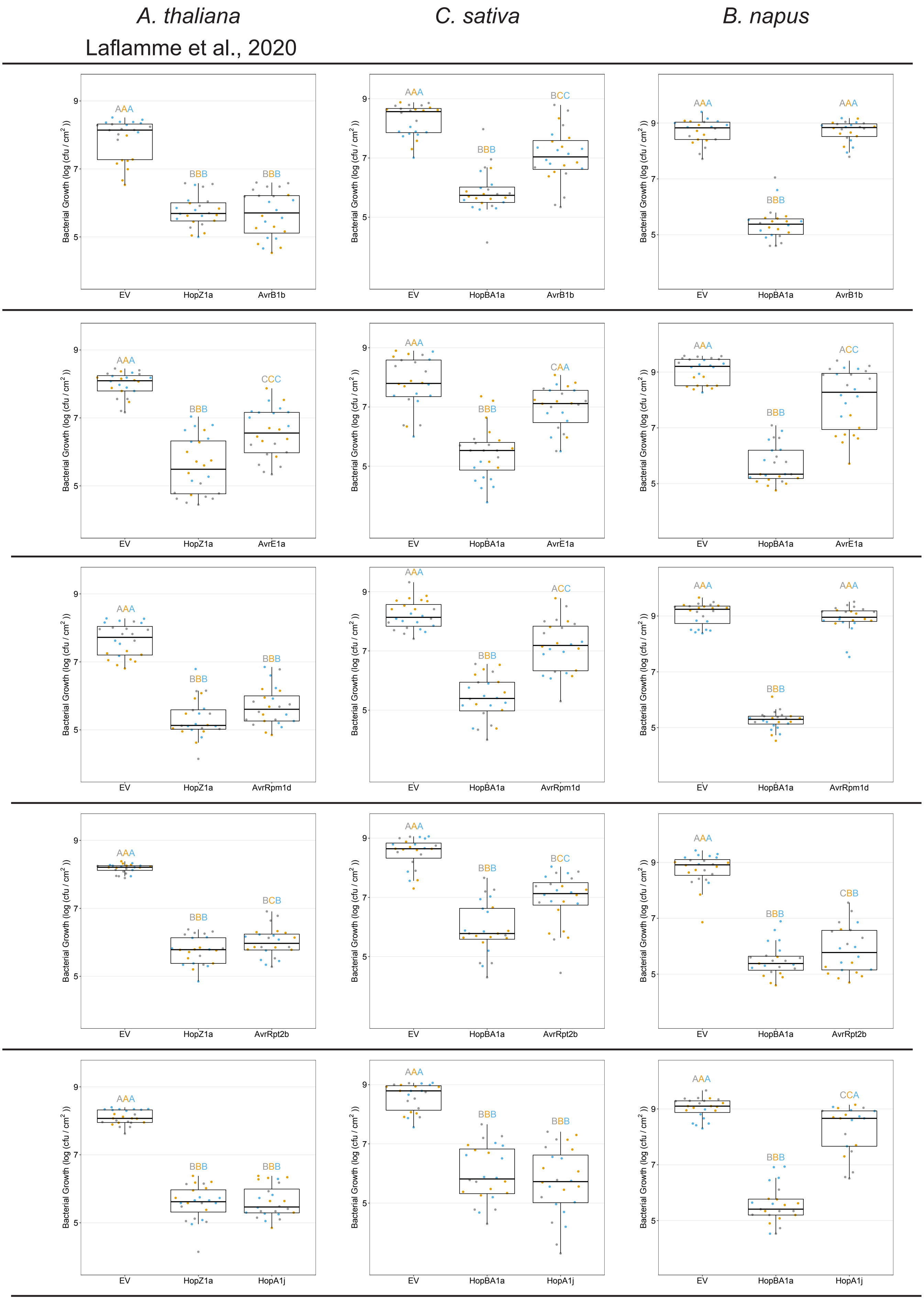
A. thaliana
Laflamme et al., 2020
C. sativa
B. napus
Bacterial Growth (log (cfu / cm2))
AAA BBB BBB
EV HopZ1a AvrB1b
AAA BBB BCC
EV HopBA1a AvrB1b
AAA BBB AAA
EV HopBA1a AvrB1b
AAA BBB CCC
EV HopZ1a AvrE1a
AAA BBB CAA
EV HopBA1a AvrE1a
AAA BBB ACC
EV HopBA1a AvrE1a
AAA BBB BBB
EV HopZ1a AvrRpm1d
AAA BBB ACC
EV HopBA1a AvrRpm1d
AAA BBB AAA
EV HopBA1a AvrRpm1d
AAA BBB BCB
EV HopZ1a AvrRpt2b
AAA BBB BCC
EV HopBA1a AvrRpt2b
AAA BBB CBB
EV HopBA1a AvrRpt2b
AAA BBB BBB
EV HopZ1a HopA1j
AAA BBB BBB
EV HopBA1a HopA1j
AAA BBB CCA
EV HopBA1a HopA1j

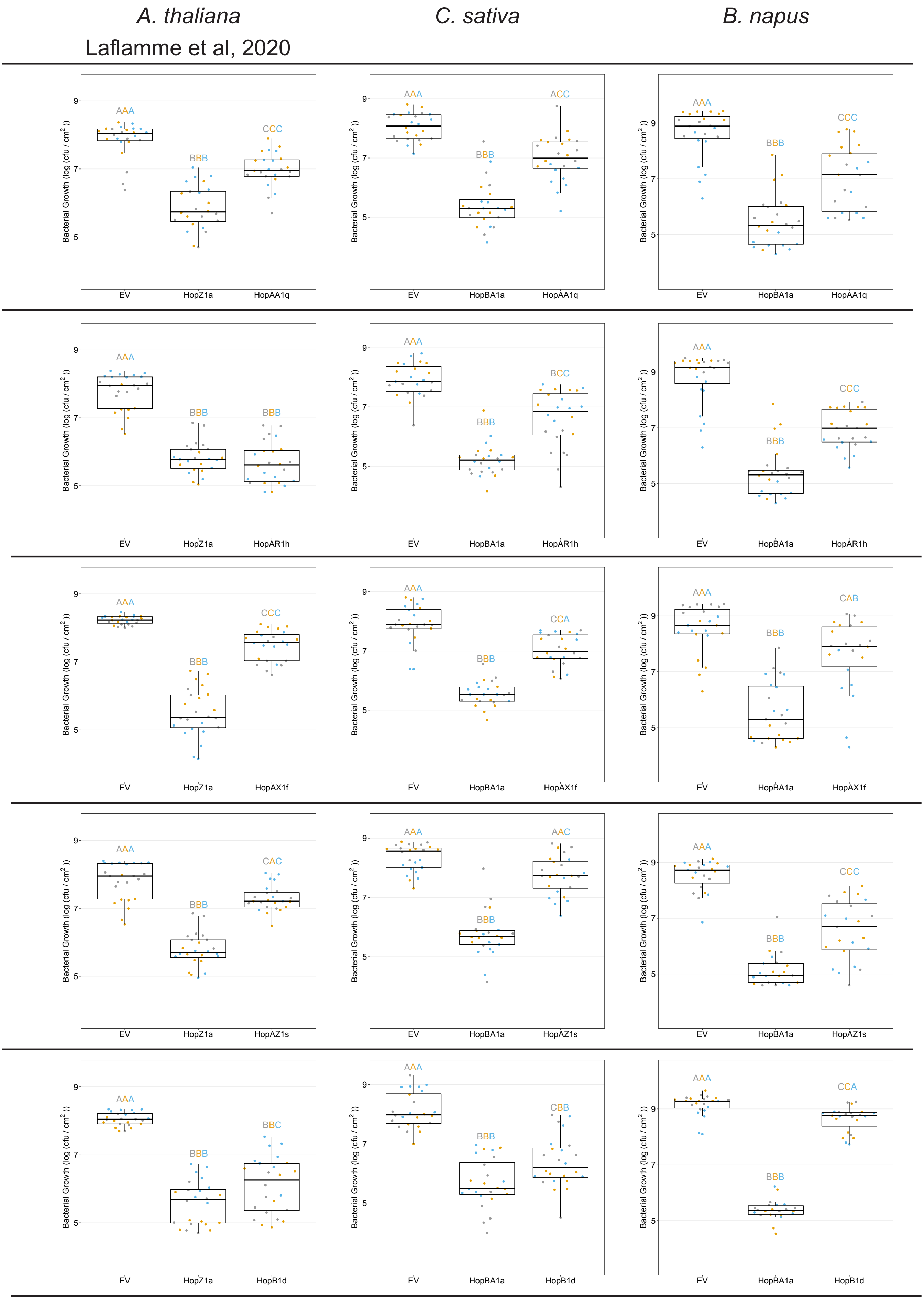
A. thaliana
Laflamme et al, 2020
C. sativa
B. napus
Bacterial Growth (log (cfu / cm²))
EV
HopZ1a
HopBA1a
HopAA1q
HopAR1h
HopAX1f
HopAZ1s
HopB1d
AAA
BBB
CCC
ACC
BCC
CCA
CAB
CAC
AAC
BBC
CBB
CCA

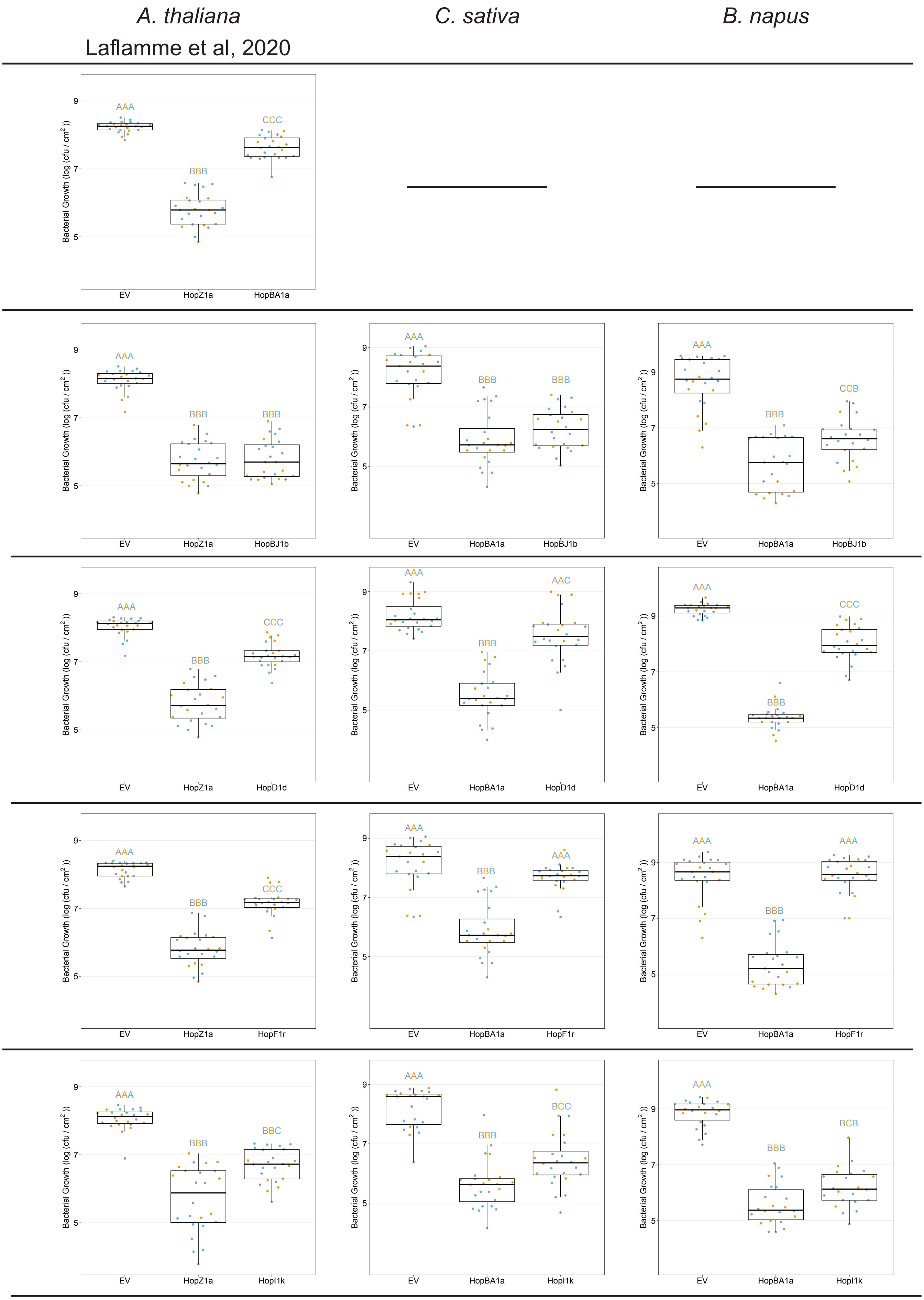

A. thaliana
Laflamme et al, 2020
C. sativa
B. napus
Bacterial Growth (log (cfu / cm^2))
9
7
5
AAA
BBB
CCC
EV
HopZ1a
HopBA1a
AAA
BBB
BBB
EV
HopZ1a
HopBJ1b
AAA
BBB
BBB
EV
HopBA1a
HopBJ1b
AAA
BBB
CCB
EV
HopBA1a
HopBJ1b
AAA
BBB
CCC
EV
HopZ1a
HopD1d
AAA
BBB
AAC
EV
HopBA1a
HopD1d
AAA
BBB
CCC
EV
HopBA1a
HopD1d
AAA
BBB
CCC
EV
HopZ1a
HopF1r
AAA
BBB
AAA
EV
HopBA1a
HopF1r
AAA
BBB
AAA
EV
HopBA1a
HopF1r
AAA
BBB
BBC
EV
HopZ1a
HopI1k
AAA
BBB
BCC
EV
HopBA1a
HopI1k
AAA
BBB
BCB
EV
HopBA1a
HopI1k

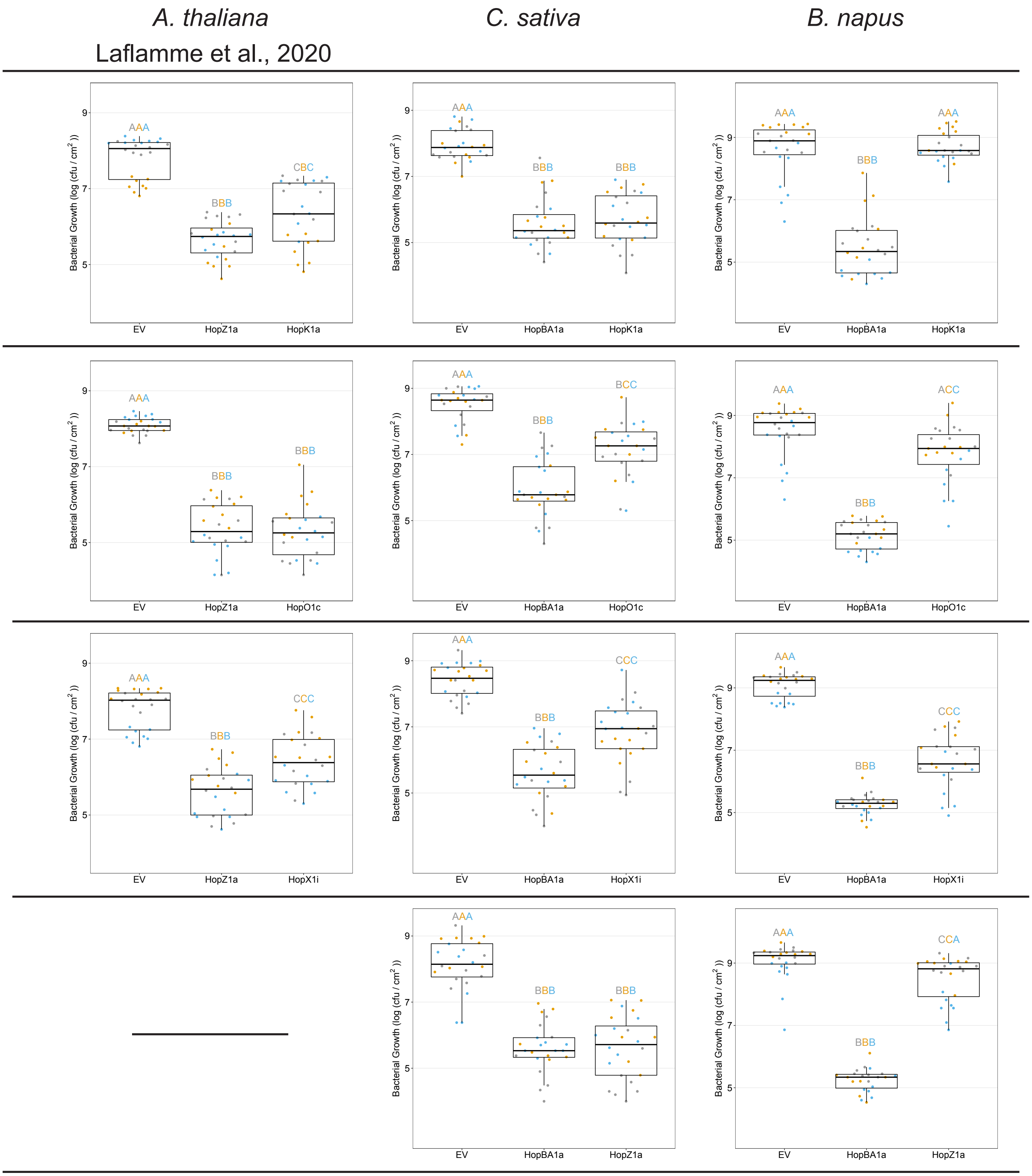

Supplemental Figure 2. Individual growth assay experimental replicates of *Pto*DC3000 strains expressing 19 representative ETI eliciting effectors in *A. thaliana* Col-0, *C. sativa* var. DH55, and *B. napus* var. Topas alongside a negative empty vector control (EV), and a positive control (HopZ1a in *A. thaliana*, HopBA1a in *B. napus* and *C. sativa*). *A. thaliana* data is from Laflamme et al., 2020. One out of three replicates of HopAZ1s on *A. thaliana* was not significantly different than EV. Boxes show the first quartile, median, and third quartile. Whiskers extend to the smallest, and largest values no further than 1.5x interquartile range from the first and third quartiles, respectively. Dots indicate individual plants, and different colours represent single experiments (n = 6, 7, or 8 plants / experiment). Letters indicate significance categories (ANOVA with post-hoc Tukey-Kramer HSD test, $P < 0.05$).

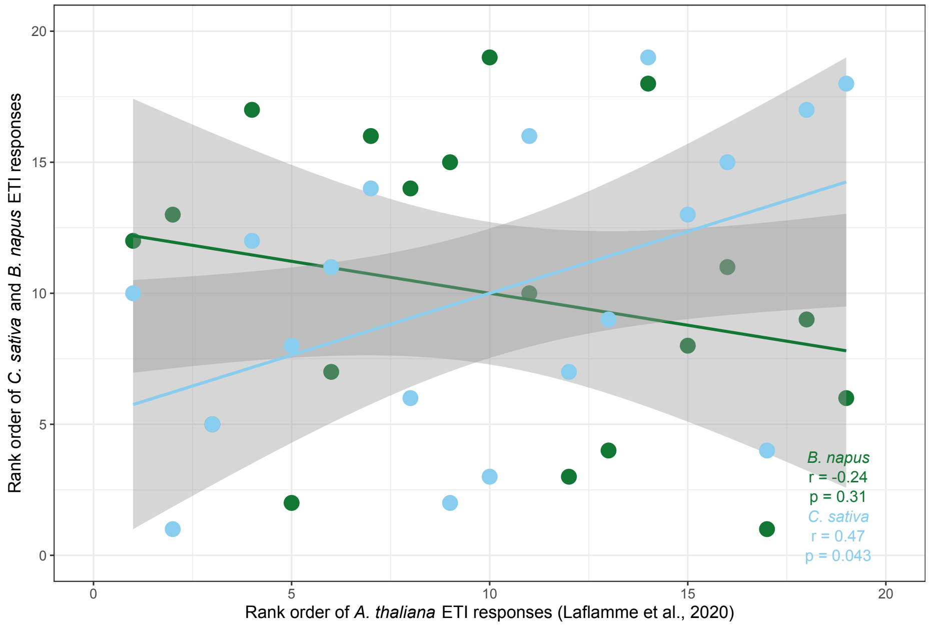

Supplemental Figure 3. Differential rank order of ETI eliciting effectors between *A. thaliana*, *B. napus*, and *C. sativa*. The rank orders of ETI responses in *B. napus* and *C. sativa* are plotted against the rank order of *A. thaliana* ETI responses (Laflamme et al., 2020) using the bacterial growth assay data presented in Figure 2, with 1 being the strongest (largest reduction in bacterial growth), and 19 being the weakest (smallest reduction in bacterial growth). Dots represent individual effectors. Blue represents *C. sativa* and green represents *B. napus*. The blue and green lines represent the linear regression models for *C. sativa* and *B. napus* data, respectively. The shaded grey areas represent the 95% confidence interval for the linear models. Spearman correlation coefficients are reported alongside the linear models and colour coded based on plant species.
