# Supplementary material for: The Arabidopsis effector-triggered immunity landscape is conserved in oilseed crops
Source: Sci Rep. 2022 Apr 20;12:6534. doi: 10.1038/s41598-022-10410-w (PMC9021255; doi:10.1038/s41598-022-10410-w)
Supplement: Supplementary file 2 — Supplementary Information 2. [file 41598_2022_10410_MOESM2_ESM.docx]

**Supplemental Tables**

**Supplemental Table 1. *A. thaliana* NLR conservation in *B. napus* and *C. sativa***

|  | **Blastp against *C. sativa* genome** | | | |  |
| --- | --- | --- | --- | --- | --- |
| *A. thaliana* NLR | RefSeq protein accession # | E-value | Percent identity | Sequence coverage | Reference |
| *RPM1*  *(AT3G07040)* | XP_010486234.1  /XP_010486237.1 | 0.0  0.0 | 85.75%  83.15% | 100%  100% |  |
| *RPS2*  *(AT4G26090)* | XP_010433556.1 | 0.0 | 87.02% | 100% |  |
|  | XP_019088486.1 | 0.0 | 86.58% | 100% |  |
|  | XP_010451179.2 | 0.0 | 87.13% | 100% |  |
| *RPS4*  *(AT5G45250)* | XP_010494613.1 | 0.0 | 86.86% | 98% |  |
|  | XP_010481621.1 | 0.0 | 86.18% | 98% |  |
| *RRS1*  *(AT5G45260)* | XP_010494612.1  (low-quality protein) | 0.0 | 83.67% | 100% |  |
|  | XP_010481619.1 | 0.0 | 84.02% | 100% |  |
| *RPS4b*  *(AT5G45060)* | XP_010441795.1 | 0.0 | 87.07% | 100% |  |
|  | XP_010496944.1 | 0.0 | 88.26% | 100% |  |
| *RRS1b (AT5G45050)* | XP_019088163.1  (low-quality protein) | 0.0 | 79.51% | 95% |  |
|  | XP_019097230.1 /XP_010494567.1 /XP_010494568.1 /XP_010494569.1 | 0.0  0.0  0.0  0.0 | 74.39%  74.39%  74.39%  74.39% | 96%  96%  96%  96% |  |
|  | XP_010494609.1 | 0.0 | 70.61% | 92% |  |
| *RPS5*  *(AT1G12220)* | XP_010435102.1 | 0.0 | 65.47% | 99% |  |
| **RPS6*  *(AT5G46470)* | XP_010445862.2 | 0.0 | 62.38% | 97% |  |
|  | XP_010494828.1 | 0.0 | 62.53% | 96% |  |
|  | XP_010482397.1 | 0.0 | 62.51% | 95% |  |
| *ZAR1*  *(AT3G50950)* | XP_010426635.1 | 0.0 | 91.74% | 100% | (Adachi et al., 2020) |
|  | XP_010515476.1 | 0.0 | 91.84% | 100% |  |
|  | XP_019099705.1 | 0.0 | 94.89% | 78% |  |
| *BAR1*  *(AT5G18360)* | XP_019090355.1 | 0.0 | 90.11% | 100% |  |
| *CAR1*  *(AT1G50180)* | XP_010479468.1  /XP_010479467.1 | 0.0  0.0 | 84.38%  82.25% | 100%  100% |  |
|  | XP_010500572.1  /XP_019099453.1  /XP_019099455.1 | 0.0  0.0  0.0 | 83.51%  83.15%  83.57% | 100%  100%  89% |  |
| *RBA1*  *(AT1G47370)* | - | - | - | - |  |
|  | **Blastp against *B. napus* genome** | | | |  |
| *A. thaliana NLR* | RefSeq protein accession # | E-value | Percent identity | Sequence coverage | Reference |
| *RPM1*  *(AT3G07040)* | XP_013685476.1 | 0.0 | 81.78% | 100% | (Grant et al., 1998) |
|  | XP_022575240.1 | 0.0 | 81.03% | 100% |  |
| *RPS2*  *(AT4G26090)* | XP_013741401.2  (low quality protein) | 0.0 | 83.64% | 100% |  |
| *RPS4*  *(AT5G45250)* | XP_022544192.1  (low quality protein) | 0.0 | 76.57% | 96% |  |
|  | XP_013712809.1 | 0.0 | 71.14% | 97% |  |
|  | XP_013734073.1 | 0.0 | 71.37% | 96% |  |
| *RRS1*  *(AT5G45260)* | - | - | - | - |  |
| *RPS4b*  *(AT5G45060)* | - | - | - | - |  |
| *RRS1b (AT5G45050)* | XP_013734065.1 | 0.0 | 67.08% | 93% |  |
|  | XP_013714869.1 | 0.0 | 64.32% | 92% |  |
| *RPS5*  *(AT1G12220)* | XP_013657483.1 | 0.0 | 76.82% | 100% |  |
| **RPS6*  *(AT5G46470)* | XP_022559634.1  /XP_022559631.1 | 0.0  0.0 | 55.59%  61.16% | 97%  81% |  |
|  | XP_013735925.2 | 0.0 | 54.52% | 97% |  |
|  | XP_013707590.1 | 0.0 | 53.98% | 95% |  |
|  | XP_013681424.2 | 0.0 | 67.51% | 72% |  |
|  | XP_022548484.1 | 0.0 | 57.01% | 82% |  |
|  | XP_013705861.1 | 0.0 | 56.36% | 94% |  |
|  | XP_022548485.1 | 0.0 | 75.72% | 55% |  |
|  | XP_022548483.1 | 0.0 | 57.13% | 79% |  |
|  | XP_013706684.2  (low quality protein) | 0.0 | 59.41% | 74% |  |
|  | XP_013724112.1 | 0.0 | 55.37% | 95% |  |
| *ZAR1*  *(AT3G50950)* | XP_013748733.1 | 0.0 | 87.66% | 100% | (Adachi et al., 2020) |
|  | XP_013697004.1 | 0.0 | 87.66% | 100% |  |
| *BAR1*  *(AT5G18360)* | XP_013740460.2 | 0.0 | 79.44% | 100% |  |
| *CAR1*  *(AT1G50180)* | XP_013735839.1 | 0.0 | 80.65% | 100% |  |
|  | XP_013714921.1 | 0.0 | 80.13% | 86% |  |
| *RBA1*  *(AT1G47370)* | - | - | - | - |  |

Note: Results reported here are from a standard protein BLAST (BLASTP) search against a non-redundant protein database with the ‘Organism’ set to *Camelina sativa* (taxid: 90675) and *Brassica napus* (taxid: 3708), respectively.

‘/’ denotes different protein isoforms.

*****Upon reciprocal BLASTP of *RPS6* hits to the *A. thaliana* genome, the top hit is *AT5G46450*, while *RPS6* (*AT5G4670*) is second. *RPS6* and *AT5G46450* share 65% protein identity and 77% protein similarity and are co-localized in the genome.
